# The c-Myc Target Glycoprotein1bα Links Cytokinesis Failure to Oncogenic Signal Transduction Pathways in Cultured Human Cells

**DOI:** 10.1371/journal.pone.0010819

**Published:** 2010-05-25

**Authors:** Qian Wu, Fengfeng L. Xu, Youjun Li, Edward V. Prochownik, William S. Saunders

**Affiliations:** 1 Department of Biology, University of Pittsburgh, Pittsburgh, Pennsylvania, United States of America; 2 Section of Hematology/Oncology, Children's Hospital of Pittsburgh, Pittsburgh, Pennsylvania, United States of America; 3 Department of Microbiology and Molecular Genetics, The University of Pittsburgh Medical Center, Pittsburgh, Pennsylvania, United States of America; 4 University of Pittsburgh Cancer Institute, Pittsburgh, Pennsylvania, United States of America; Roswell Park Cancer Institute, United States of America

## Abstract

An increase in chromosome number, or polyploidization, is associated with a variety of biological changes including breeding of cereal crops and flowers, terminal differentiation of specialized cells such as megakaryocytes, cellular stress and oncogenic transformation. Yet it remains unclear how cells tolerate the major changes in gene expression, chromatin organization and chromosome segregation that invariably accompany polyploidization. We show here that cancer cells can initiate increases in chromosome number by inhibiting cell division through activation of glycoprotein1b alpha (GpIbα), a component of the c-Myc signaling pathway. We are able to recapitulate cytokinesis failure in primary cells by overexpression of GpIbα in a p53-deficient background. GpIbα was found to localize to the cleavage furrow by microscopy analysis and, when overexpressed, to interfere with assembly of the cellular cortical contraction apparatus and normal division. These results indicate that cytokinesis failure and tetraploidy in cancer cells are directly linked to cellular hyperproliferation via c-Myc induced overexpression of GpIbα.

## Introduction

The transition from the restrained and controlled growth of normal cells to the accelerated and dysregulated growth of cancer cells requires multiple changes, including enhancement of the signaling pathways controlling division and survival. But additional changes not directly related to increased proliferation usually accompany these cellular alterations. These include genetic instability (GI), aneuploidy, and centrosome amplification, all of which are associated with a loss of genomic integrity [1], [2], [3], [4]. The reason the two phenotypes of enhanced growth and GI so often appear together is currently unknown. It is commonly believed that GI imparts a “mutator” phenotype to the cancer cells, increasing the genetic diversity necessary for the selection of mutant clones with enhanced growth and survival [5]. But since GI is strongly associated with senescence and apoptosis [6], [7], [8], it is unclear how cells tolerate the deleterious effects of GI long enough for these cellular evolutionary steps to occur. It is also unclear whether the mechanisms that cause polyploidization are directly related to the signals that cause enhanced growth or whether they are an indirect consequence of elevated proliferation rates.

Two key, and related, genomic destabilizing events that are believed to contribute to cancer are tetraploidization, the doubling of the chromosome number, and centrosomal amplification, which increases the number of microtubule organizing centers in the cell. It has long been believed that tetraploidy is an important intermediate in cellular transformation, as cancer cells typically have increased chromosome numbers [1], [9], [10]. More recently, tetraploidy has been directly linked to tumorigenesis in mice [11], [12], and centrosome amplification has been linked to tumor growth in flies [13]. But in both of these model systems, tetraploidy and centrosome amplification were artificially induced by mechanisms not directly associated with carcinogenesis. The root cause of tetraploidy and centrosome amplification in cancer cells therefore remain mostly uncharacterized.

One of the classic oncoproteins that enhance growth and proliferation of cancer cells is the transcription factor c-Myc. Highly overexpressed in malignant cells, c-Myc modifies a variety of processes including cell proliferation, differentiation, survival, GI and metabolism [14]. Overexpression of c-Myc is sufficient for acute transformation of immortalized rodent cell lines, allowing them to become tumorigenic in immunocompromised mice. One of the many targets of c-Myc transcriptional regulation is GpIbα, a subunit of the von Wilebrand factor receptor (vWFR) that is responsible for the adhesion, aggregation and activation of platelets upon binding to damaged epithelium [15], [16]. Recent data shows that GpIbα has additional functions that are independent of the blood-clotting pathway but are linked to c-Myc mediated transformation and induction of GI. These include reducing the need for growth factors, inhibiting apoptosis, causing DNA and nuclear damage, promoting tetraploidy and transforming immortalized cells [12], [17]. GpIbα is also necessary to promote tetraploidy by c-Myc activation and is sufficient to do this in the absence of overt c-Myc deregulation [17].

To understand in more detail the role of GpIbα in promoting GI, we have identified the genomic-destabilizing events associated with GpIbα overexpression. We show here that GpIbα localizes to the cleavage furrow of dividing primary cells and that overexpression of GpIbα interferes with the correct localization of key divisional proteins at the cleavage furrow associated with failure of cytokinesis or cell division. These observations provide the first direct mechanistic link between stimulation of cell proliferation and transformation, via the c-Myc signaling pathway, and the genomic destabilizing events of polyploidization and centrosomal amplification.

## Results

### GpIbα overexpression caused failure of cytokinesis

**Figure 1:**
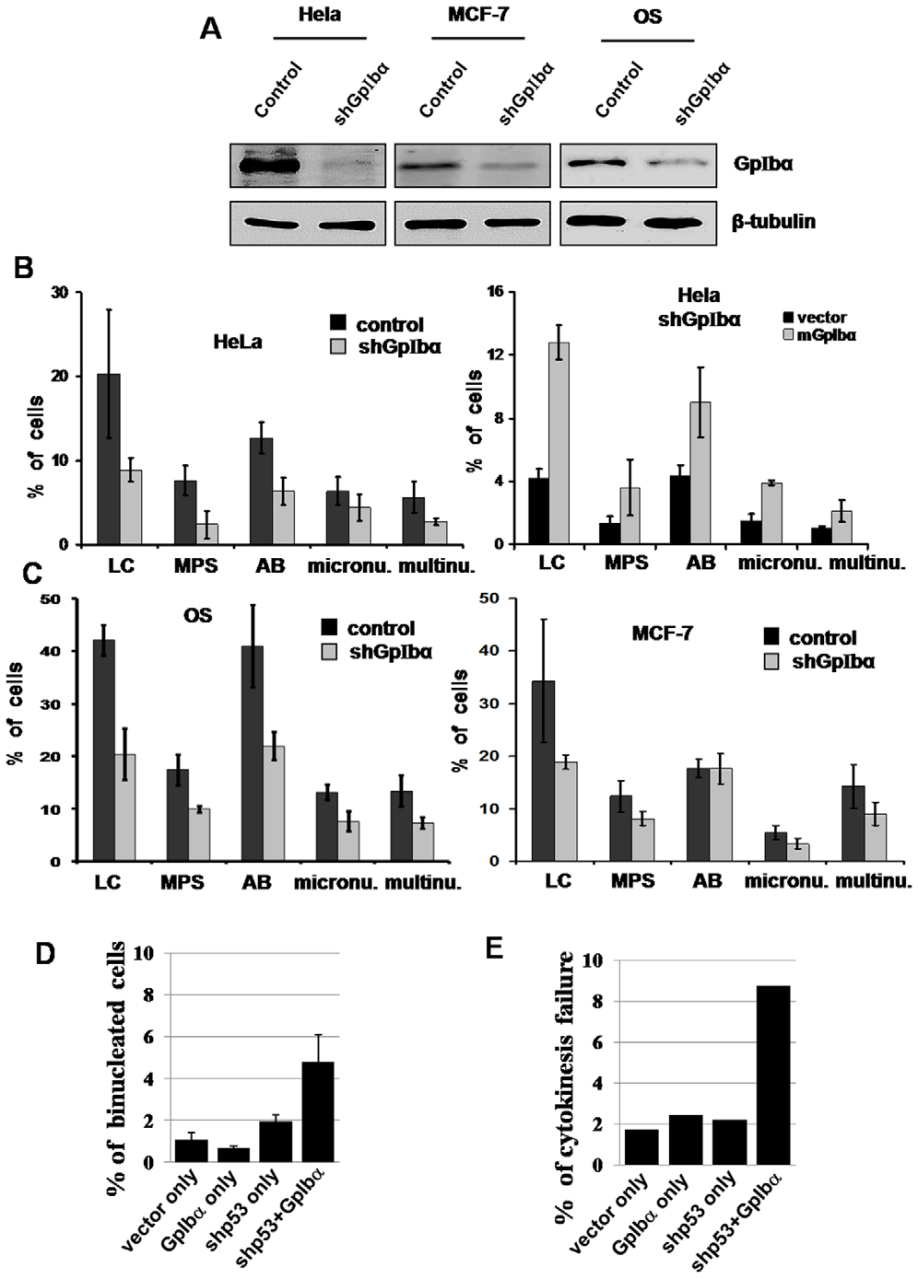
Knockdown of endogenous GpIbα in cancer cells reduces the frequencies of mitotic and cytokinesis defects, while overexpression of GpIbα in immortalized noncancer cells increases the frequencies of binucleation and cytokinesis failure. (A) Immunoblots of HeLa, MCF-7 and OS whole cell extracts from shRNA GpIbα knockdown cultures and respective controls. (B) Frequencies of mitotic and cytokinesis defects in HeLa cells were significantly decreased after GpIbα knockdown and enforced overexpression of a shRNA-resistant GpIbα (mGpIbα) restored most of the mitotic defects (n>100 cells per sample). (C) The percentages of OS and MCF7 cells demonstrating mitotic and cytokinesis defects were reduced by GpIbα knockdown (n>100 cells per sample). In both (B) and (C): Examined mitotic defects include: lagging chromosomes (LC), multipolar spindles (MPS), anaphase bridges (AB), micronuclei (micron.) and multinucleation (>1 nuclei, multin.). (D) HFF-hTERT cells with stable knockdown of p53 and/or overexpression of GpIbα were stained with DAPI and the frequency of binucleated cells were determined by fluorescent microscopy (n = 300–500 cells per sample). Note that cancer cells occasionally have more than two nuclei and cells with two or more nuclei were categorized in Figure 1B and 1C as “multinucleated”. HFF-hTERT cells very rarely had more than two nuclei and cells with two nuclei were categorized in Figure 1D as “binucleated”. (E) Frequency of cytokinesis failure in the same cell lines as in D determined by DIC live-cell imaging. Divisional failure was markedly stimulated by overexpression of GpIbα in a p53 deficient background. At least 50 dividing cells were analyzed in each category in E. In B, C and D, data and error bars represent mean and standard deviation of at least three different experiments.

GpIbα is widely overexpressed in a variety of tumors and tumor cell lines and GpIbα overexpression gives rise to tetraploidy in primary human foreskin fibroblasts (HFF; [12], [17]. To determine if GpIbα overexpression was the cause of nuclear amplification in cancer cells, GpIbα was stably knocked down by a short hairpin RNA in HeLa, OS osteosarcoma, and MCF7 breast cancer cell lines (Figure 1A and Figure S1A and S1B). The frequency of multinucleates (an example is shown in Figure S1C, a common result of cytokinesis failure), was markedly (p<0.05) reduced in HeLa and OS cell lines, and moderately (p<0.1) reduced in the MCF7 cell line (Figure 1B and C). In addition, the frequency of multipolar spindles (MPS, an example is shown in Figure S1D), a hallmark of centrosome amplification, was also significantly (p<0.05) reduced in HeLa and OS cells, and moderately (p<0.1) reduced in MCF7 cells. Many other mitotic and cytokinesis defects including anaphase bridges, lagging chromosomes and micronuclei, demonstrated similar trends after GpIbα knockdown in tested cancer cells (Figure 1B and C), showing that overexpression of GpIbα is a significant cause of cytokinesis failure and mitotic defects in malignant cells. Furthermore, these results were validated by expressing a murine shRNA-resistant GpIbα (mGpIbα) in HeLa-shGpIbα cells and as expected, we observed increases in mitotic and cytokinesis defects compared with control shGpIbα cells (vector alone) showing the specificity of the knockdown phenotype (Figure 1B).

We next examined whether overexpression of GpIbα was sufficient to impair cytokinesis in noncancer primary cells. A series of HFF cells stably immortalized with human telomerase (hTERT) were used for this study, including HFF-vector (stably transfected with empty vector), HFF-shp53 (p53 stably knocked down by a short hairpin RNA), HFF+GpIbα (stably overexpressing GpIbα), and HFF-shp53+GpIbα (stably overexpressing GpIbα with p53 knockdown) [12]. The frequency of binucleates in interphase cells increased markedly when GpIbα was overexpressed, but only in a p53 knockdown background (Figure 1D), consistent with previous findings [12]. To confirm that the binucleation was due to cytokinesis failure, we observed the division of >300 cells by live-cell differential interference contrast (DIC) microscopy. Cytokinesis failure was seen in approximately 2% of the vector-alone cells, shp53 or GpIbα overexpressing cells. However, failure of division increased by >4-fold in HFF-shp53+GpIbα cells (Figure 1E and Movies S1 and S2). These results show that overexpression of GpIbα is sufficient to lead to cytokinesis failure in immortalized primary cells lacking p53 and provide an explanation for the increased multinucleation and ploidy of cancer cells.

### GpIbα colocalizes with F-actin at the cleavage furrow during cytokinesis

**Figure 2:**
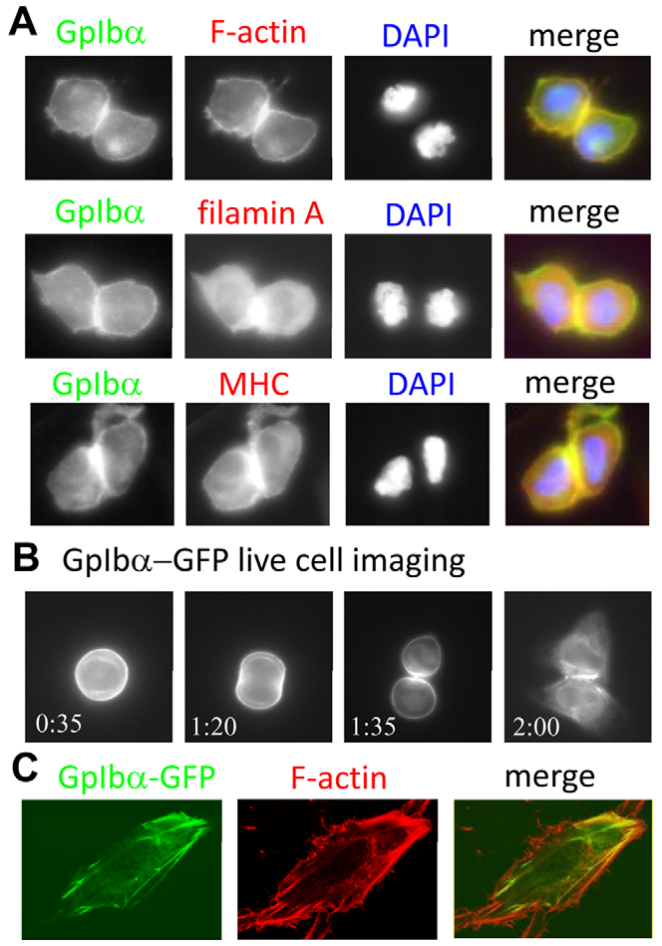
GpIbα co-localizes with F-actin, filamin A and MHC. (A) Co-localization between GpIbα and F-actin, MHC and filamin A at the cleavage furrow was observed in HFF-vector cells by double immunofluorescence. DAPI staining was used to confirm that the cells were in telophase. The merge combines anti-GpIbα in green and the indicated second primary antibody in red. (B) GpIbα localization during mitosis via live cell imaging using HeLa cells transiently transfected with GpIbα-GFP. Time stamp is hrs∶mins shown from the start of imaging. These stills are from Movie S3. (C) GpIbα-GFP in interphase cells partially co-localized with F-actin at the cell cortex. HeLa cells were transiently transfected with GpIbα-GFP, fixed and analyzed by confocal microscopy. F-actin was visualized with rhodamine-phalloidin (Cytoskeleton).

To determine if GpIbα plays a role in cell division, its localization was evaluated in dividing HFF-hTERT cells by immunofluorescence. In late mitosis, endogenous GpIbα concentrated at the contractile ring in the midzone of the dividing cell, co-localizing with F-actin, filamin A, and myosin heavy chain (MHC, Figure 2A). This is notably different from the ER localization of GpIbα described previously in interphase cells where GpIbα is distributed diffusely throughout the cytoplasm and in association with the ER ([18] and Figure S2A). As a control, another membrane-associated marker, CD44, did not concentrate at the cleavage furrow (Figure S2B), showing the cleavage furrow enrichment is specific to a subset of membrane-associated proteins. To examine the changing dynamics of GpIbα positioning, GFP was fused to the C-terminus of GpIbα and the fusion protein was transiently expressed in HeLa cells, which tolerated the expression better than primary cells. As observed with immunolocalization in primary cells, GpIbα-GFP concentrated at the cleavage furrow in mitotic HeLa cells (Figure 2B and Movie 3), thus confirming that GpIbα associated with contractile structures of the cell during division. However, unlike primary cells GpIbα-GFP staining was only observed in a fraction of the dividing HeLa cells (discussed further below). More diffuse cytoplasmic staining was seen in interphase cells, consistent with the previously described ER localization [12]. GpIbα-GFP also partially colocalized with F-actin fibers near the cell cortex of interphase cells (Figure 2C) indicating an association with the actin cytoskeletal in nondividing cells.

### GpIbα overexpression causes mislocalization of filamin A, F-actin, MHC and RhoA from the contractile ring

**Figure 3:**
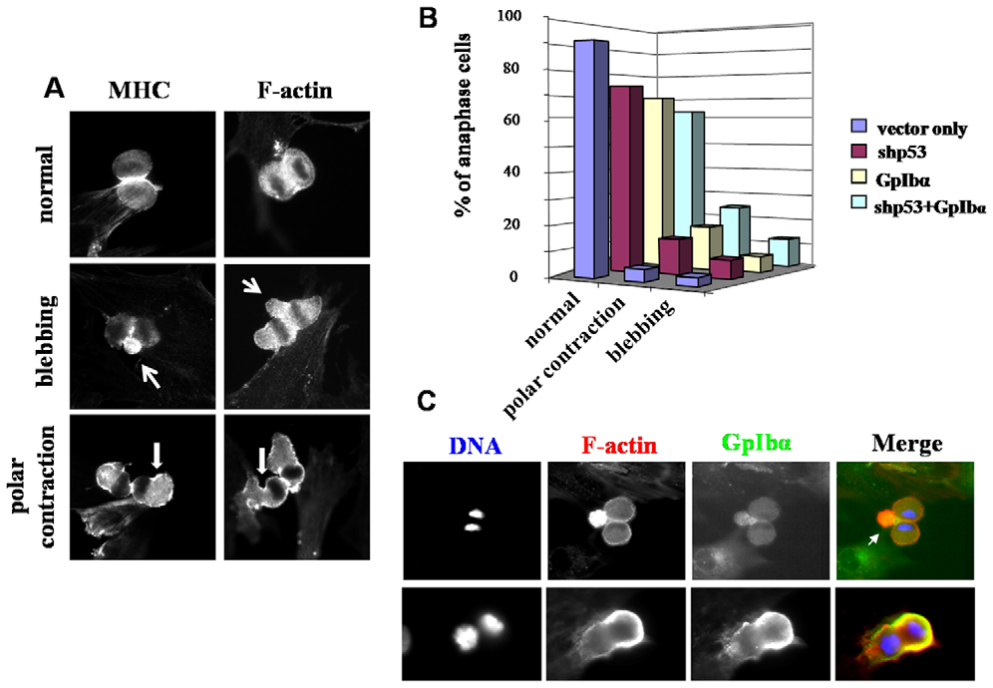
Abnormal cortical contraction observed in cells overexpressing GpIbα. (A) The localization of MHC and F-actin was visualized in HFF-shp53+GpIbα cells by immunofluorescence. The abnormal divisional morphologies of blebbing and polar contraction were observed and are marked by arrows. (B) Quantification of the frequency of the different categories of abnormal morphologies from (A). (C) Blebbing (top panel, arrow) and polar contraction (bottom panel) structures in HFF-shp53+GpIbα cells during cytokinesis contained both actin and GpIbα, as seen by double immunofluorescence.

As we documented real-time divisional failure in GpIbα-overexpressing cells (Figure 1E), we also observed defects of the cortical structure of the cells consistent with abortive contraction. One such defect was membrane blebbing seen by immunofluorescence with antibodies to MHC or F-actin (Figure 3A, arrows). Similar blebbing structures have been seen with failed cytokinesis from other sources [19], [20]. We also noted polar contraction, defined as cortical contraction and F-actin and myosin accumulation outside of the cleavage furrow during division (Figure 3A). Both blebbing and polar contraction are abnormal features and were only rarely observed in HFF-vector cells, but were found ∼30% of the HFF-shp53+GpIbα cells (Figure 3B). When these aberrant divisional structures and processes formed, they typically contained both F-actin and GpIbα, as shown in Figure 3C for blebbing in the top panel and polar contraction in the bottom panel. Small molecule inhibition (ML-7) of the signaling protein myosin light chain kinase blocked cytokinesis but did not lead to blebbing or polar contraction confirming that these are symptoms of contractile or abscission defects and are not found in all cases of cytokinesis failure. These observations suggest that the actomyosin contractile cytoskeletal organization and function in dividing cells is defective following overexpression of GpIbα.

**Figure 4:**
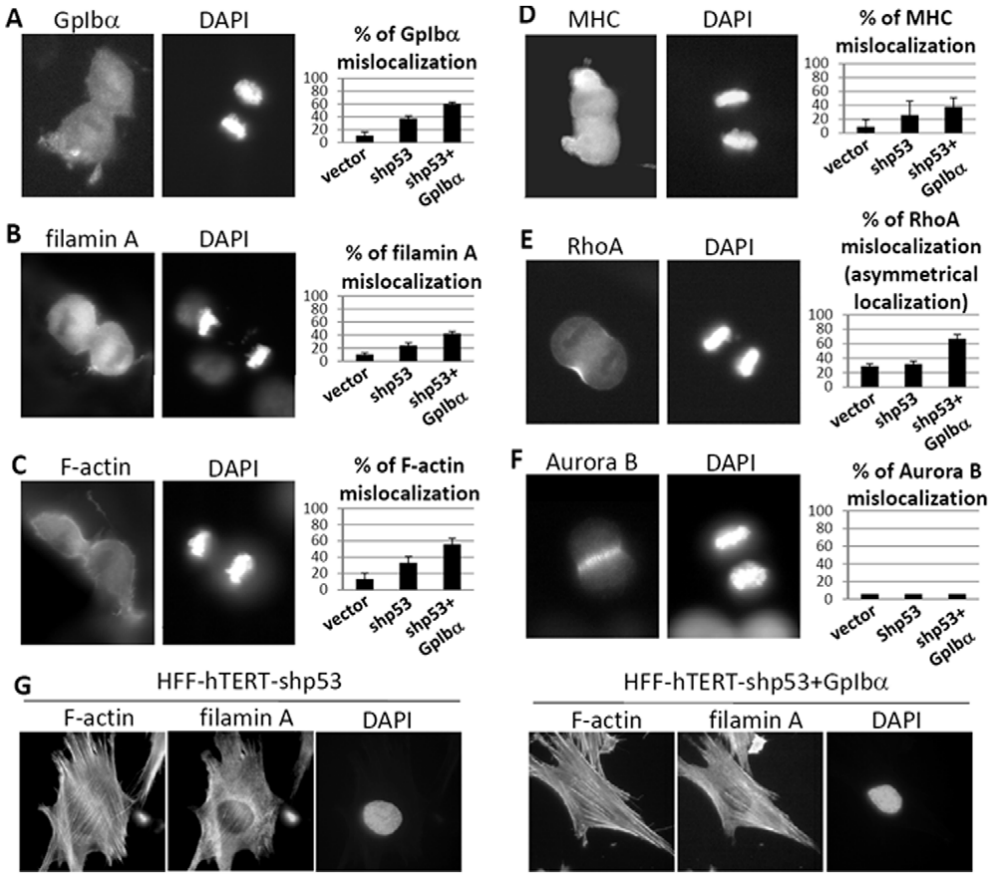
GpIbα-overexpression causes mislocalization of some cytokinesis-related proteins. (A–E). Left panels: Immunofluorescence revealed that GpIbα, filamin A, F-actin, and MHC were frequently absent and RhoA asymmetrically localized at the cleavage furrow in HFF-shp53+GpIbα cells during cytokinesis. Right panels: The means and standard deviations of the protein mislocalization (n>100 cells per sample). (F) Aurora B localization was not affected by GpIbα overexpression or p53 deletion (n = 100 cells per sample). (G) Interphase localizations of F-actin and filamin A were not affected by GpIbα overexpression. For panels A–E, the p value between HFF-vector and the HFF-shp53+GpIbα cells are each <0.05 by an unpaired two-tailed Student's t-test.

To examine in more detail the molecular nature of the divisional defect, the localization of a variety of divisional proteins were examined in GpIbα-overexpressing cells. Several key cytokinesis proteins were missing in a subset of the dividing cells. Surprisingly, GpIbα itself was missing from the contractile rings in about 60% of anaphase HFF-shp53+GpIbα cells (Figure 4A). (Comparable results were observed by live cell imaging of HeLa cells stably transfected with the GpIbα-GFP described above.) Similarly, filamin A, F-actin and MHC were also often absent from the cleavage furrow of dividing HFF-shp53+GpIbα cells, while the interphase localizations of filamin A and F-actin were not affected (Figure 4B, C, D and G). We believe that these cytokinesis protein mislocalizations were related to the abnormal divisional structures described above. Fully 80.8% of HFF-shp53+GpIbα cells with abnormal filamin A localization showed blebbing during division. Filamin A deficiencies have been previously shown to cause blebbing during cell locomotion [21]. The cytokinesis activator, RhoA, was often asymmetrically localized in HFF-shp53+GpIbα cells, with stronger staining at one edge of the furrow (Figure 4E). In contrast, the mitotic signaling kinase Aurora B was normally positioned at the cleavage furrow in dividing HFF-shp53+GpIbα cells (Figure 4F), showing that the mislocalization was specific to a subset of cytokinesis proteins.

**Figure 5:**
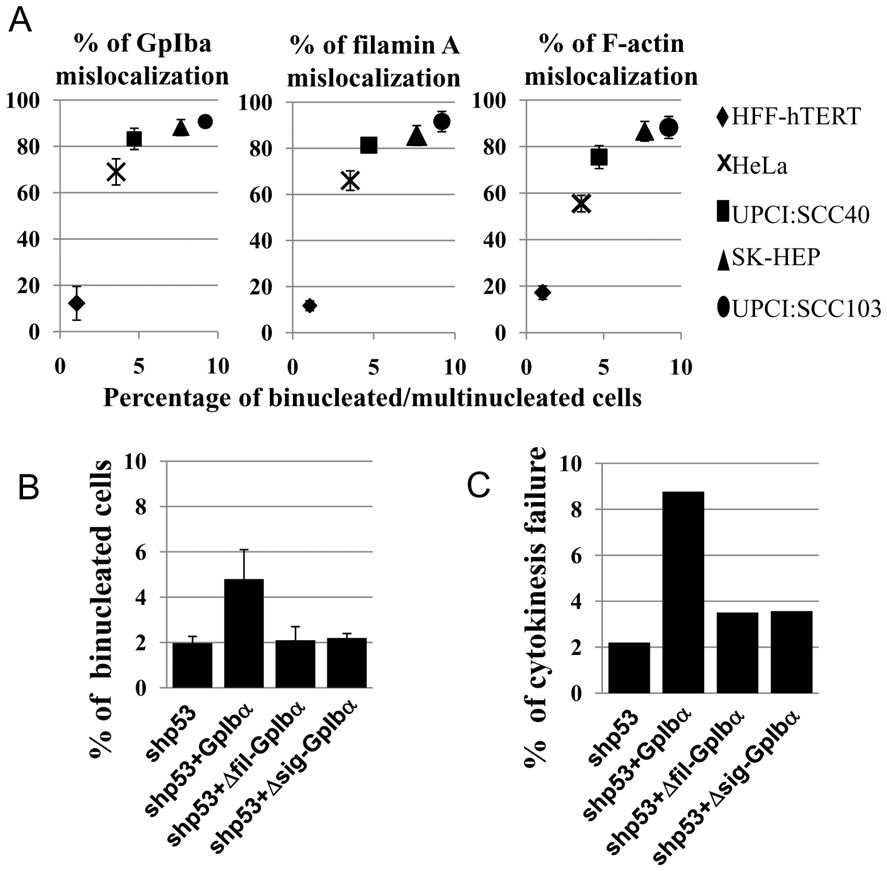
GpIbα overexpression is correlated with multinucleation in tumor cells and requires the filamin A-binding domain and signal peptide to efficiently induce cytokinesis failure. (A) Correlation between GpIbα, filamin A, and F-actin mislocalization during cytokinesis and percentage of binucleation in different cancer cell lines compared with a control HFF-vector cell line. Protein localization was determined by immunofluorescence and the frequency of binucleates by DAPI staining. All of the data are averages of at least three independent experiments, with at least 100 mitotic cells or 500 interphase cells counted in each category. (B) The frequency of binucleation in HFF-shp53 cells overexpressing GpIbα mutants with deletions of the filamin A binding domain (shp53+Δfil-GpIbα) or the ER signal sequence (shp53+Δsig-GpIbα) is less than cells overexpressing the full length protein (shp53+GpIbα; p<0.01) and similar to HFF-shp53 controls (p>0.3). Means and standard deviation of three experiments with ∼500 cells counted per experiment are shown. (C) The frequency of cytokinesis failure seen by DIC microscopy in HFF-shp53 cells overexpressing GpIbα mutants with deletions of the filamin A binding domain or the ER signal sequence is less than cells overexpressing the full length protein and similar to HFF-shp53 controls (n = 50–100 cells imaged for each cell line).

The above studies showed that GpIbα overexpression resulted in the mislocalization of key divisional proteins from the cleavage furrow of dividing primary cells. We next determined if the localization of the same proteins was compromised in cancer cells. The distribution of GpIbα, F-actin and filamin A during cytokinesis in four cancer cell lines including HeLa, liver adenocarcinoma SK-HEP-1 and oral squamous cell carcinoma derived UPCI∶SCC40 and UPCI∶SCC103 was examined by immunofluorescence. All of the tested cancer cell lines showed frequent mislocalization of these divisional markers, similar to HFF-shp53+GpIbα cells in Figure 4, and we observed a correlation between the frequency of binucleated/multinucleated cells and the frequency of GpIbα, F-actin and filamin A mislocalization (Figure 5A). These observations show that the mislocalization of cytokinesis proteins seen with GpIbα-overexpression in primary cells can also be seen in malignant cells and is associated with failure of division. To determine if a reduction of GpIbα was able to reverse the marker mislocalization in cancer cells, we compared localization of filmain A and F-actin in HeLa cells before and after shRNA knockdown of GpIbα (Figure S3). In both cases a small decrease was observed, but this was significant only for Filamin A (p = 0.016). We interpret these results to indicate that GpIbα overexpression does play a role in cytokinesis failure and divisional protein mislocalization in cancer cells, but that additional unknown factors may be acting to interfer with cytokinesis protein localization.

### Signal peptide and filamin A binding domains of GpIbα are indispensible for GpIbα-overexpression mediated cytokinesis failure

We further explored which domains of GpIbα were important for inhibition of cytokinesis. One region of interest was the filamin A binding domain to test the significance of interactions of GpIbα with this actin modifying protein. A second region of interest was the signal peptide domain to determine if transit through the secretory pathway was required for overexpressed GpIbα to inhibit cytokinesis. Cellular fractionation was used to verify the mutant lacking the signal peptide was unable to localize to the ER (Figure S4). When overexpressed, these mutants were much less effective at increasing the binucleation frequency observed in DAPI-stained HFF-hTERT cells (Figure 5B), or the frequency of cytokinesis failure viewed by live-cell DIC microscopy (Figure 5C). These results indicated that interference with cytokinesis required that the overexpressed GpIbα be capable of entering the ER secretory pathway and binding to filamin A, thus further supporting the conclusion that GpIbα overexpression inhibits cytokinesis by interfering with the cortical F-actin filament network. These findings are consistent with previous observations showing that GpIbα-induced GI was abrogated by loss of either the filamin A-binding domain or signal peptide of GpIbα [17].

### GpIba overexpression is responsible for transformation-related features of cancer cells

It is conventionally believed that tetraploidy resulting from cytokinesis failure is an intermediate step towards tumorigenesis [11], [12]. As our data have demonstrated that GpIbα overexpression led to cytokinesis failure and tetraploidization, we next investigated whether GpIbα overexpression was required for the elevated growth rates and tumorigenic properties of cancer cells.

**Figure 6:**
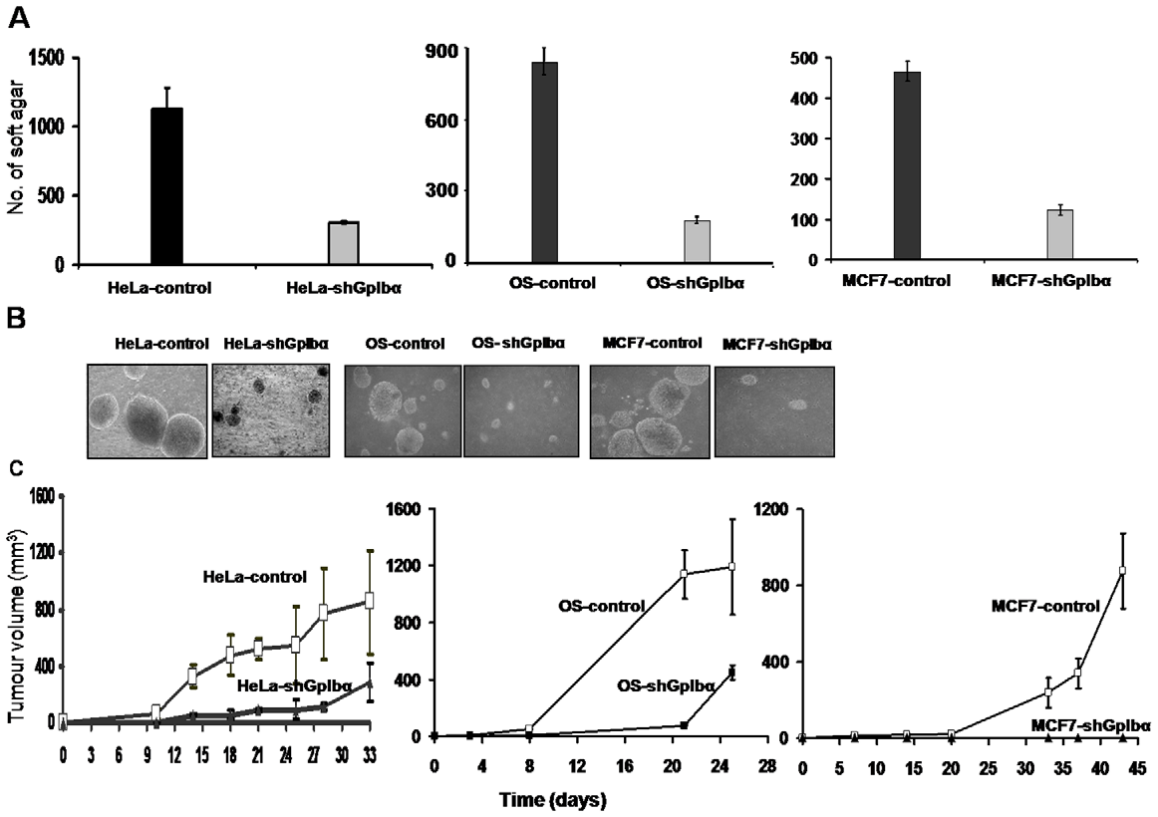
Loss of transformed phenotypes by shGpIba cell lines. (A) Reduced clonogenic growth of shRNA cells Equivalent numbers of shRNA and control cells were plated in soft agar as previously described [12], [21]. After 12–14 days of growth, the total number of macroscopically visible colonies was evaluated in triplicate cultures. The values shown depict the average number of clones +/−1 S.E. (B) Photomicrographs of representative colonies from each of the cell types. (C) Reduced tumorigenicity of shGpIbα cell lines. Groups of nude mice (3–4 animals/group) were inoculated subcutaneously with ca. 107 of the indicated cell types and then monitored weekly for evidence of tumor formation. Note the significant growth impairment of tumors originating from shGpIbα-expressing tumor cell lines.

When endogenous GpIbα in tumor cells was knocked down, we observed markedly reduced clonogenicity in soft agar compared with controls, even when high serum concentrations were maintained or the periods of culture were extended to compensate for possible reduced rates of proliferation (Figure 6A and data not shown). Moreover, the colonies that did arise from shRNA cells were invariably of much smaller overall size (Figure 6B). These results were validated by expression of a shRNA resistant murine mGpIbα which restored enhanced growth showing the specificity of the shRNA knockdown (Figure S5). We therefore conclude that anchorage-independent growth of the tested cancer cells lines was profoundly influenced by endogenous GpIbα levels.

Furthermore, when we tested each shRNA cell line and its control counterpart by inoculating immunocompromised nu/nu mice with equivalent numbers of cells, we found that in all three cases, endogenous GpIbα knockdown resulted in a significant impairment of tumor growth (Figure 6C). Collectively, we conclude that GpIbα overexpression is responsible for hyperproliferation and tumorigenesis of the tested cancer cells.

## Discussion

This study makes two advances towards understanding GI in tumor cells. The first is to establish a mechanism for cytokinesis failure in cancer cells and the second is to link cytokinesis failure mechanistically with enhanced growth and proliferation via c-Myc.

Increased GpIbα expression, a common feature of tumor cells [18], was shown to contribute to cytokinesis failure in the tested cancer cell lines. Furthermore, we were able to establish a working model for how tetraploidy originates in cancer cells by overexpression of GpIbα and p53 inhibition in immortalized primary cells. In these cells, cytokinesis failure was accompanied by the appearance of abnormal contractile structures and mislocalization of essential divisional proteins, including F-actin, filamin A and RhoA. Similar mislocalization of F-actin, filamin A and GpIbα could be seen in tumor-derived cells and in each case was correlated with the appearance of multinucleation, a feature of cytokinesis failure. These results show for the first time that the genomic destabilizing event of cytokinesis failure in cancer cells can be defined at the molecular level and reproduced with similar phenotypes in primary cells.

The phenotypes from GpIbα overexpression in primary cells were markedly more severe in the absence of p53. It has been observed previously that loss of this genomic checkpoint protein facilitates c-Myc induced tetraploidy [22] and promotes survival of the cells following genomic damage from GpIbα overexpression [12]. Similarly, the loss of p53 may be required here to bypass cellular checkpoints that otherwise inhibit abnormal cytokinesis in primary cells, although this explanation alone is insufficient to explain the protein mislocalization we see from p53 knockdown. Additionally, while a reduction of GpIbα led to some normalization of cytokinesis protein localization in cancer cells, GpIbα, F-actin and filamin A remained mislocalized in many HeLa cells after knockdown of GpIbα demonstrating that other factors also interfere with cytokinesis protein positioning in these cells.

Inhibition of cytokinesis by GpIbα overexpression requires filamin A binding and we have shown that filamin A localizes to the mammalian cleavage furrow in a GpIbα-dependent manner. Previously, filamin A was found in chick embryonic cells at the cleavage furrow [23]. Filamin A is known to bind to GpIbα as part of the vWFR signaling pathway [24], [25], and we propose that GpIbα-filamin A interactions are also important for cell division. Filamin A homodimerizes at a flexible hinge and crosslinks polymerized actin into a 3-dimensional gel, promoting F-actin networks rather than the anti-parallel arrays associated with contractile fibers in skeletal sarcomeres [21], [26]. It may therefore seem surprising that filamin A function would be important for contractile mechanisms in cytokinesis. However, the contractile forces at the cleavage furrow have also been proposed to result from disordered actin arrays [27], [28], and we propose that filamin A crosslinking of F-actin may be an important part of that process. Filamin A binds RhoA [29] that was also mislocalized in GpIbα-overexpressing cells. It is controversial whether RhoA activity is essential for formation of the cleavage furrow [19], [30], but it is important to activate formin leading to actin polymerization during division [31] and is thought to be required for cortical contraction [32]. Deficiency in RhoA also leads to blebbing as we observe in GpIbα-overexpressing cells [19]. Thus, GpIbα-induced interference with filamin A and RhoA positioning/function may explain the actomyosin and contractile deficiencies we observe in GpIbα-overexpressing cells.

The observation that a source of cytokinesis failure in cancer cells is a target of the c-Myc pathway that stimulates growth and division, could help explain the linkage between oncogenic transformation and tetraploidy. Since both pathways are activated concurrently, by the same molecular changes, it is logical that they would be found together in the same cells. Furthermore, this may help explain how the cell tolerates the mitotic disruption of centrosome and chromosome amplification. We propose that a tight phenotypic linkage between the cause of cytokinesis failure and stimulated cell growth and proliferation offsets the intrinsic cost of abnormal division and polyploidy on cell survival. This may allow the cells to thrive despite the selective disadvantage of GI. In this model, we interpret cytokinesis failure and tetraploidy to be linked to enhanced cellular proliferation, not as a direct consequence of proliferation, but because both processes are induced by c-Myc activation and GpIbα overexpression.

We have demonstrated a role for GpIbα in cytokinesis and the increases in ploidy common in cancer cells. Previously, GpIbα was known for its role as a platelet- and megakaryocyte-specific cell surface receptor [15], [16]. Its presence on other cell lineages, where there is little or no expression of the other vWFR components, raises interesting questions regarding the origin and functionality of this subunit. It is possible that the original function of GpIbα was related to cytokinesis, and only later did it evolve into other specialized roles in platelets and megakaryocytes. It is also possible that some of its known functions in platelets and megakaryocytes could be related to a role in cytokinesis. During maturation megakaryocytes undergo endomitosis, several rounds of mitosis without cell division [33]. Recent evidence shows that endomitosis in megakaryocytes involves a failure of the contractile ring [34]. Both endomitosis and aborted division in cancer cells may utilize similar pathways involving GpIbα, although further investigations will be required to test this hypothesis.

## Materials and Methods

### Cell lines and cell culture

HFF-vector, HFF-GpIbα, HFF-shp53, HFF-shp53+GpIbα cells were generated as described [12]. HFF-shp53+Δsig-GpIbα and HFF-shp53+Δfil-GpIbα cells were generated using the same method described in reference 12, with the plasmids constructed in reference 18. All HFF cells were maintained in DMEM medium (Sigma) supplemented with 2 mM L-Glutamine (Sigma) and 10% fetal bovine serum (FBS; Sigma). HeLa cells (ATCC) were maintained in DMEM medium supplemented with 10% FBS. UPCI∶SCC40 and UPCI∶SCC103 cell lines are gifts from Dr. Susanne M. Gollin (University of Pittsburgh). Both of the two UPCI∶SCC cell lines and liver adenocarcinoma cell line SK-HEP-1 (ATCC) were maintained in MEM (Sigma) supplemented with 10% FBS, 2 mM L-Glutamine and 1% non-essential amino acids (Invitrogen). All cells were cultured at 37°C with 5% CO_2_.

### GpIbα shRNA knockdown

Retroviral vectors (pHUSH) encoding human GpIbα shRNA 29-mers and a puromycin-selectable cassette (Cat. Numbers TR12692) were obtained from Origene, Inc. (Rockville, MD) and 100 units/ml Penicillin G + 100 µg/ml Streptomycin as previous described [35]. Retroviral transfections were performed in Phoenix-A cells as previously described using Superfect (Qiagen, Chatsworth, CA; [36]. Phoenix A supernatants were then harvested daily beginning 48 hr after transfection with retroviral vectors, filtered by passage through 0.45 µM filters (Millipore, Bedford, NY) and applied to cancer cell line monolayers for 24 hr in the presence of 8 µg/ml Polybrene (Sigma-Aldrich, St. Louis, MO). After 2–3 applications, cells were cultured in fresh, virus-free medium for 48 hr followed by selection in puromycin-containing medium (1 µg/ml; Sigma-Aldrich). Puromycin-resistant colonies were pooled for all subsequent studies and were intermittently maintained in puromycin-containing medium.

### Plasmid and DNA transfections

2×10^5^ cells were seeded on 22×22 mm glass coverslips (VWR) in 6-well plates and incubated with pre-warmed OPTI-MEM (Invitrogen) medium. After six hours, cells were transfected with 2 µg of plasmid using 6 µl of the FuGENE6 transfection reagent (Roche Diagnostics) following the manufacture's protocol. Fresh medium was added 12 hours later. Cells were examined 24–48 hours after transfection.

### Immunofluorescence

Cells on coverslips were fixed in 4% paraformaldehyde at room temperature and washed in PBS. 0.1% Triton X-100 was used to permeabilize the cells and 1.5% BSA/PBS was used as blocking solution. Various primary antibodies were used including rAb-MHC (Sigma, 1∶500), mAb-actin (Cytoskeleton, 1∶100), mAb-filamin (a gift from Dr. Nakamura, Translational Medicine Division, Brigham and Women's Hospital, Boston, MA, 1∶500), ratAb-Gp1ba (Emfret, 1∶100), mAb-RhoA (Santa Cruz Biotechnology, 1∶100), and mAb-CD44 (BD Pharmingen, 1∶1000). All primary antibodies were diluted in the blocking solution and incubated for 30 minutes at room temperature. Fluorescent labeled goat anti-rabbit or anti-mouse or anti-rat IgG (Invitrogen, 1∶500) were diluted in the blocking solution as secondary antibodies. After PBS wash, cells were incubated with the desired secondary antibody for 30 minutes at room temperature followed by staining with 4,6-diamidino-2-phenylindole (DAPI) at 1 µg/ml (Sigma) for 5 minutes. The coverslips were mounted and examined by Olympus BX60 epifluorescence microscope with 100× oil immersion objectives. Hamamatsu Argus-20 CCD camera was used to capture the images. Confocal microscopy was performed using Nikon Eclipse E800 (Nikon) with BioRad Radiance 2000 system.

### Live microscopy analysis

2×10^5^ cells were seeded on 35 mm glass-bottom Petri dishes (MatTek Corporation) and subject to live cell imaging either after transfection with desired DNA plasmids as described above or without any treatment. Cells were videoed while being maintained at 37°C with a moisturized-warm air microscope chamber (Life Imaging Services, Reinach, Switzerland). DIC microscopy and epifluorescence microscopy were performed on Nikon Eclipse TE2000-U inverted microscope with Coolsnap HQ digital camera (Roper Scientific Photometrics). Images were taken and analyzed using MetaMorph software (Molecular Devices).

### Immunoblotting

Cellular fractionation proteins were loaded onto 10% SDS-PAGE gels and separated by electrophoresis and transfer onto PVDF membranes (Biorad, Hercules, CA). Antibodies against CD44 (BD, San Jose, CA), calnexin (Stressgen, Ann Arbor, MI), actin (cytoskeleton, Denver, CO), GAPDH (Cell signaling, Danvers, MA), and GFP (Abcam, Cambridge, MA) or GP1Bα (Emfret Analytics & CO.) were all diluted in 5% milk/TBST and used as primary antibodies. Membranes were incubated with the primary antibody overnight at 4°C. After 15 minute TBST wash, membranes were incubated with anti-mouse or anti-rabbit IgG-HRP-linked secondary antibodies (Amersham, GE Healthcare, UK) diluted in 5% milk/TBST for 1 hour at room temperature. Results were visualized using enhanced chemiluminescent kit (Pierce, Rockford, IL).

### *In vivo* tumorigenesis studies

All studies were reviewed and approved by The University of Pittsburgh's Institutional Animal Use and Care Committee. 6–8 wk old nu/nu mice were purchased from Harland Laboratories (Indianapolis, IN). They were housed under sterile, germ-free conditions with 12 hr day-night cycles and were allowed access to feed and water *ad libitum*. Animals were inoculated subcutaneously in the flank with 10^7^ tumor cells that had been trypsinized, washed, and immediately resuspended in PBS. They were monitored at least twice weekly and tumor volumes were calculated as previously described [12].

### Real time qRT-PCR-RNA

Extraction was performed as previously described followed by treatment with TurboDNAse as recommended by the supplier (Ambion, Austin TX)[37]. qRT-PCR was performed using a QuantiTect SYBR Green RT-PCR kit according to the directions of the supplier (Qiagen) and as previously described. Primers for the detection of human GpIbα were identified using the Primer3 program (www.frodo.wi.mit.edu/). They consisted of the sequences between nt 781 (forward) and 91 (reverse) of the transcript (GenBank Accession no. NM_000173) and were synthesized by IDT (Coralville, IA). Cycling was performed on triplicate samples on a Roche LightCycler 2.0 apparatus (Roche Diagnostics, Indianapolis, IN) and values were adjusted to those obtained for GAPDH qRT-PCR reactions performed in parallel.

## Supporting Information

Figure S1 Knockdown of GpIbα in cancer cell lines HeLa, OS, MCF7 and representative images of binucleation and multipolar spindles, two types of mitotic defects in cancer cell lines. (A) Immunofluorescence analysis of each cell line (HeLa, OS, MCF7) showing reduced expression of GpIbα in shRNA lines versus control lines. As a control, cells were also stained with calnexin (green) and with DAPI (blue) as previously described [21]. (B) qRT-PCR analyses of each cell line showing levels of GpIbα transcripts after adjusting to GAPDH levels. Each point represents the average of triplicate samples +/−1 S.E. (C) Representative samples of DAPI of chromatin and phalloidin staining of F-actin used to count the frequency of binucleates/multinucleates are shown. Mononucleate (mononuc.) and binucleate (binuc.) examples are indicated. (D) Representative immunofluorescence images with microtubule and NuMA centrosomal staining to determine spindle polarity are shown. Bipolar and multipolar (MPS) examples are indicated.
(2.40 MB TIF)

Figure S2 Control experiments for GpIbα localization in dividing cells. (A) GpIbα localizes to the cleavage furrow separate from the ER marker calnexin, as shown by immunofluorescence. (B) HFF-hTERT cells were examined by fluorescence microscopy after staining with antibodies to CD44 and GpIbα. GpIbα, but not CD44, localizes to the cleavage furrow showing that only specific membrane-associated proteins are concentrated in the divisional plane of the cell.
(0.99 MB TIF)

Figure S3 Changes in filamin A, and F-actin localization after GpIbα knockdown in HeLa cells. The frequency of protein mislocalization following stable shGpIbα transfection in HeLa cells was determined by immunofluorescence. A modest, but statistically significant, restoration of filamin A localization was observed indicating that GpIbα overexpression contributes to mislocalization in these cancer cells. But other unknown factors are apparently also controlling cytokinesis protein mislocalization in malignant cells. Standard error about the means is shown.
(0.07 MB TIF)

Figure S4 GpIbα mutants lacking signal peptide is truly defective in localizing to ER. Top two panels: Western blotting shows successful cellular fractionation to separate ER proteins and non-ER proteins. Calnexin: an ER protein marker. Middle two panels: wild-type GpIbα was found in both ER and non-ER fractions, while signal peptide-deleted GpIbα was only found in non-ER fraction. Bottom panel: loading control beta-tubulin.
(0.13 MB TIF)

Figure S5 Restoration of GpIbα expression rescues the phenotype of shRNA tumor cell lines. Two shGpIbα cell lines were transfected with a murine GpIbα expression vector or the empty parental vector. (A) Stably transfected clones were pooled and subjected to immunoblotting to verify the re-expression of GpIbα. (B) Each of the four cell lines from (A) was seeded at 104 cells/well in 6 well plates. The following day, the medium was replaced with fresh medium containing 1% FBS. Total viable counts were then determined on triplicate well at the indicated times afterwards. (C) 4×103 cells of each line were plated in soft agar and allowed to grow as anchorage-independent colonies for 14 days at which time the average no. of colonies/well was determined on triplicate samples. (D) Typical appearance of soft agar colonies after 14 days.
(0.37 MB TIF)

Movie S1 An example of cytokinesis failure. DIC live-cell imaging microscopy was used to visualize the cell division. Note: a binucleated (tetraploid) cell was generated after cytokinesis failure.
(1.48 MB MOV)

Movie S2 An example of successful cytokinesis.
(1.61 MB MOV)

Movie S3 The localization of GpIbα in cell division. GpIbα was enriched at the cell cortex, then at the division site and finally it was enriched at the cleavage furrow and persisted through the completion of cytokinesis. The cell division was recorded with a fluorescence live-cell imaging microscope using HeLa cells transfected with GpIbα-GFP.
(8.28 MB MOV)
