# Supplementary figures and images for: The c-Myc Target Glycoprotein1bα Links Cytokinesis Failure to Oncogenic Signal Transduction Pathways in Cultured Human Cells
Source: PLoS One. 2010 May 25;5(5):e10819. doi: 10.1371/journal.pone.0010819 (PMC2876040; doi:10.1371/journal.pone.0010819)

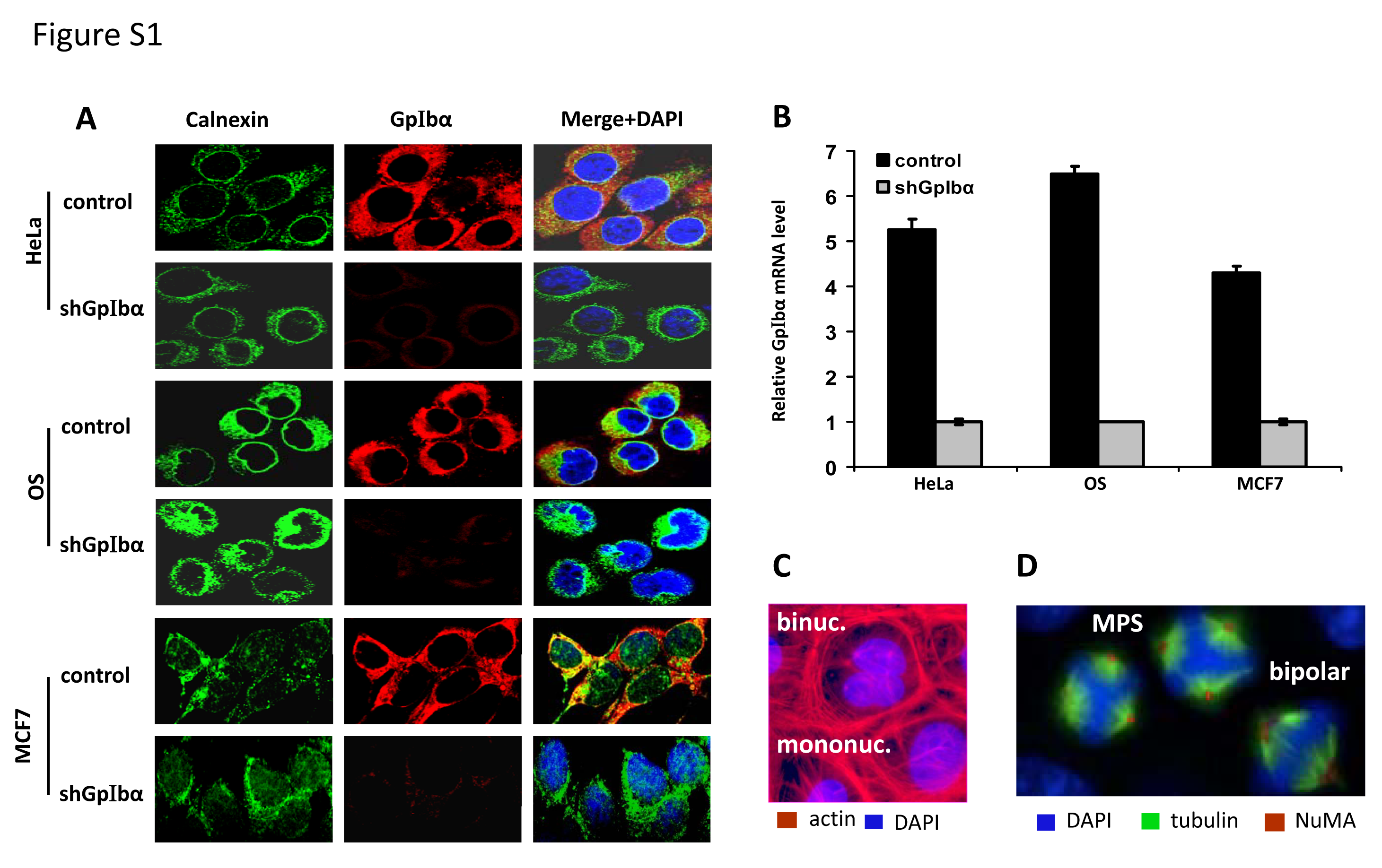

Supplement: Figure S1 — Knockdown of GpIbα in cancer cell lines HeLa, OS, MCF7 and representative images of binucleation and multipolar spindles, two types of mitotic defects in cancer cell lines. (A) Immunofluorescence analysis of each cell line (HeLa, OS, MCF7) showing reduced expression of GpIbα in shRNA lines versus control lines. As a control, cells were also stained with calnexin (green) and with DAPI (blue) as previously described [21]. (B) qRT-PCR analyses of each cell line showing levels of GpIbα transcripts after adjusting to GAPDH levels. Each point represents the average of triplicate samples +/−1 S.E. (C) Representative samples of DAPI of chromatin and phalloidin staining of F-actin used to count the frequency of binucleates/multinucleates are shown. Mononucleate (mononuc.) and binucleate (binuc.) examples are indicated. (D) Representative immunofluorescence images with microtubule and NuMA centrosomal staining to determine spindle polarity are shown. Bipolar and multipolar (MPS) examples are indicated. (2.40 MB TIF) [file pone.0010819.s001.tif]

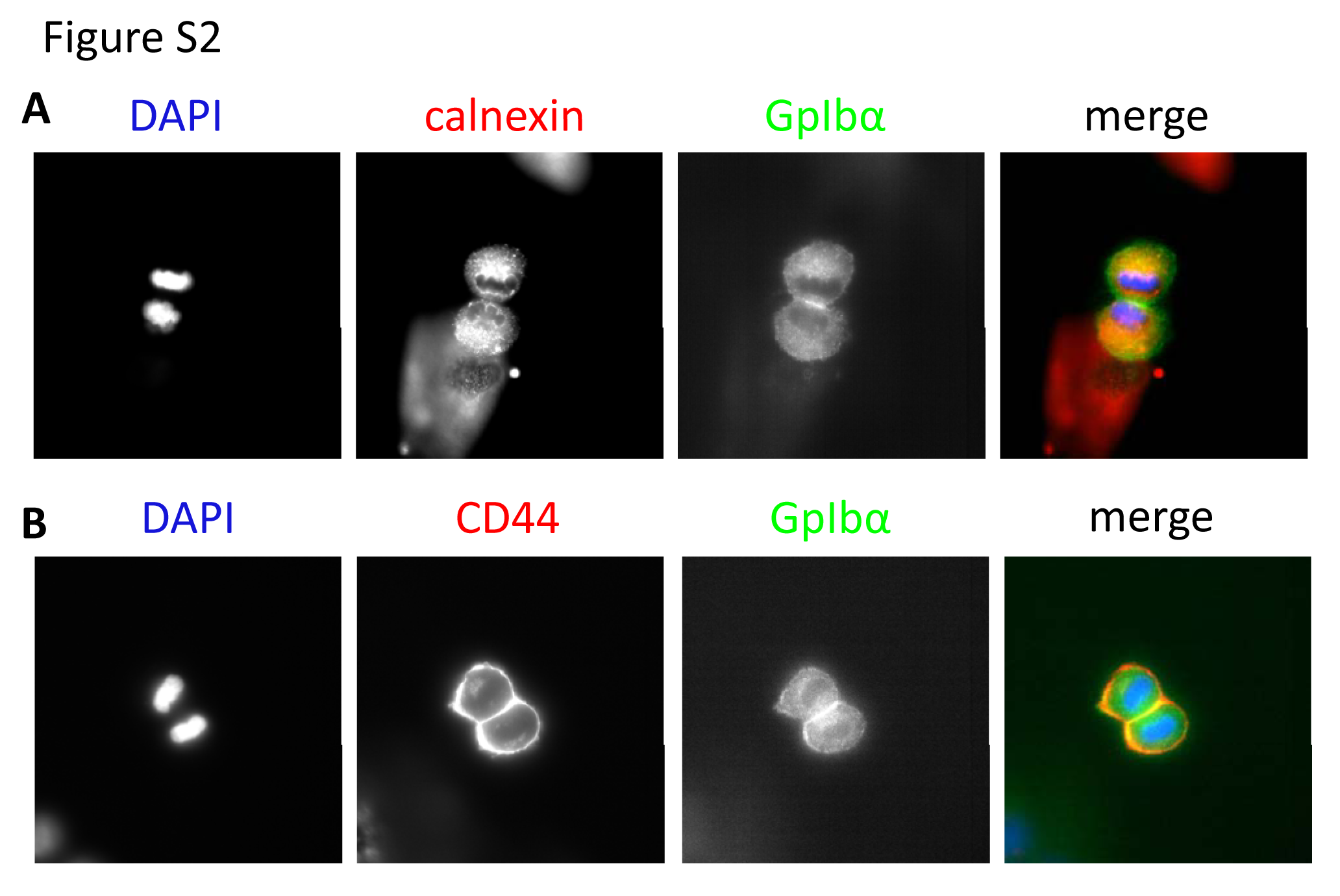

Supplement: Figure S2 — Control experiments for GpIbα localization in dividing cells. (A) GpIbα localizes to the cleavage furrow separate from the ER marker calnexin, as shown by immunofluorescence. (B) HFF-hTERT cells were examined by fluorescence microscopy after staining with antibodies to CD44 and GpIbα. GpIbα, but not CD44, localizes to the cleavage furrow showing that only specific membrane-associated proteins are concentrated in the divisional plane of the cell. (0.99 MB TIF) [file pone.0010819.s002.tif]

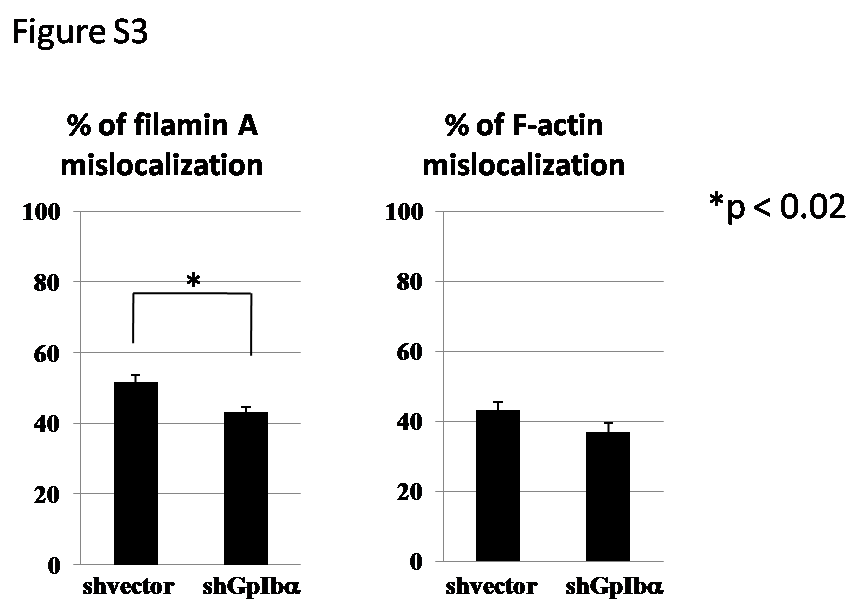

Supplement: Figure S3 — Changes in filamin A, and F-actin localization after GpIbα knockdown in HeLa cells. The frequency of protein mislocalization following stable shGpIbα transfection in HeLa cells was determined by immunofluorescence. A modest, but statistically significant, restoration of filamin A localization was observed indicating that GpIbα overexpression contributes to mislocalization in these cancer cells. But other unknown factors are apparently also controlling cytokinesis protein mislocalization in malignant cells. Standard error about the means is shown. (0.07 MB TIF) [file pone.0010819.s003.tif]

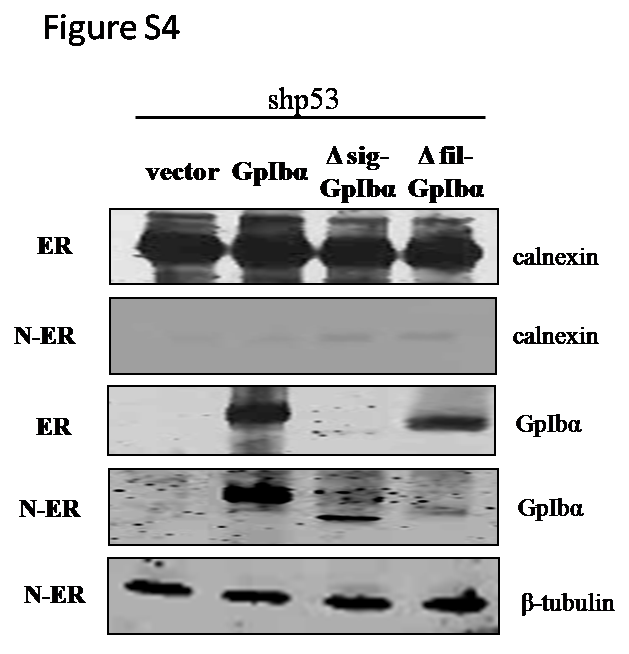

Supplement: Figure S4 — GpIbα mutants lacking signal peptide is truly defective in localizing to ER. Top two panels: Western blotting shows successful cellular fractionation to separate ER proteins and non-ER proteins. Calnexin: an ER protein marker. Middle two panels: wild-type GpIbα was found in both ER and non-ER fractions, while signal peptide-deleted GpIbα was only found in non-ER fraction. Bottom panel: loading control beta-tubulin. (0.13 MB TIF) [file pone.0010819.s004.tif]

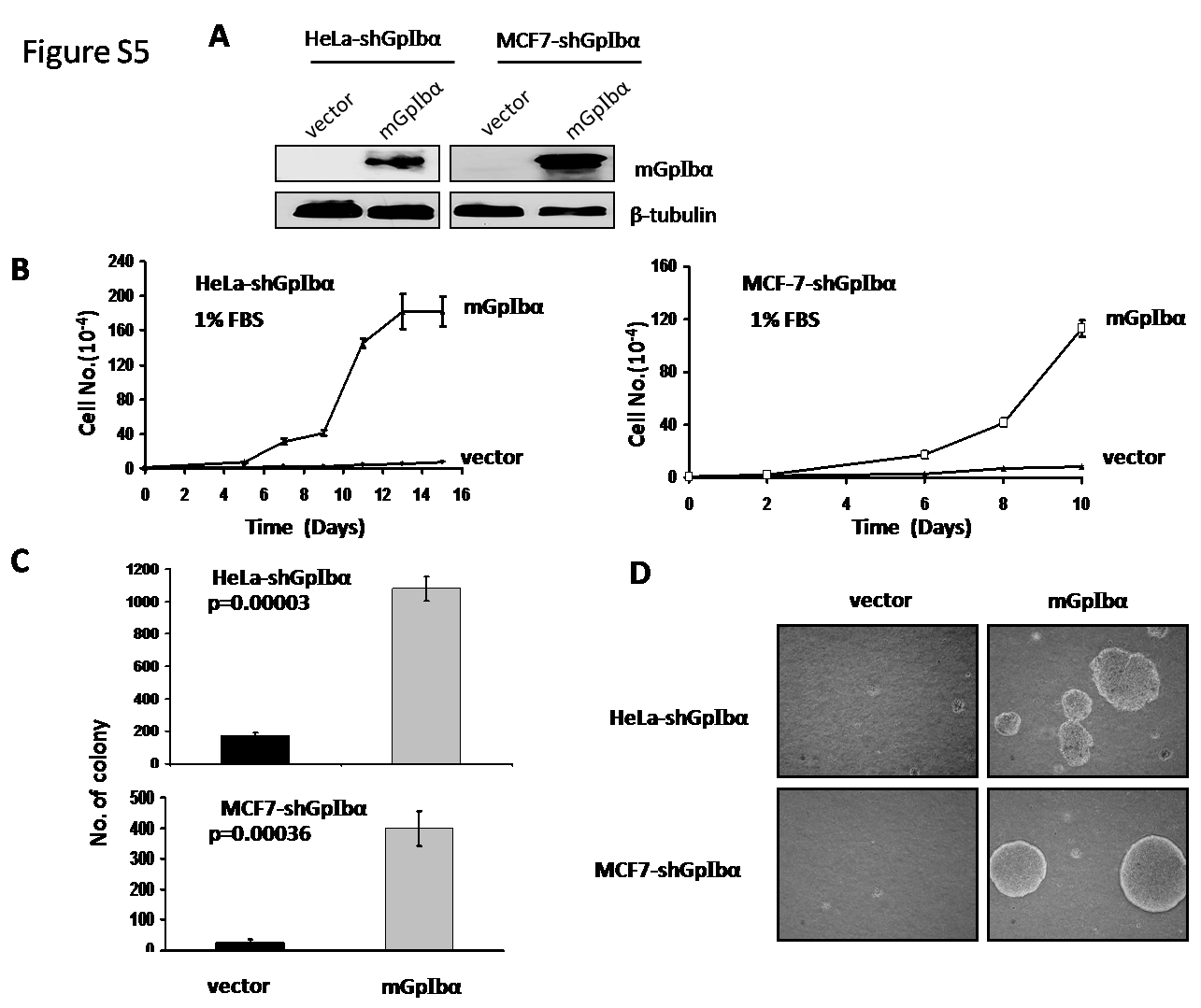

Supplement: Figure S5 — Restoration of GpIbα expression rescues the phenotype of shRNA tumor cell lines. Two shGpIbα cell lines were transfected with a murine GpIbα expression vector or the empty parental vector. (A) Stably transfected clones were pooled and subjected to immunoblotting to verify the re-expression of GpIbα. (B) Each of the four cell lines from (A) was seeded at 104 cells/well in 6 well plates. The following day, the medium was replaced with fresh medium containing 1% FBS. Total viable counts were then determined on triplicate well at the indicated times afterwards. (C) 4×10^3^ cells of each line were plated in soft agar and allowed to grow as anchorage-independent colonies for 14 days at which time the average no. of colonies/well was determined on triplicate samples. (D) Typical appearance of soft agar colonies after 14 days. (0.37 MB TIF) [file pone.0010819.s005.tif]
